# Does early-onset multiple sclerosis differ from adult-onset form in Iranian people

**Published:** 2010

**Authors:** Fereshteh Ashtari, Vahid Shaygannejad, Ziba Farajzadegan, Ali Amin

**Affiliations:** a Department of Neurology, School of Medicine, Isfahan University of Medical Sciences, Isfahan, Iran; b Isfahan University of Medical Sciences, Isfahan, Iran; c Isfahan Medical Education Research Center, Isfahan, Iran; d School of Medicine, Isfahan University of Medical Sciences, Isfahan, Iran

**Keywords:** Multiple Sclerosis, Optic Neuritis, Adulthood

## Abstract

**BACKGROUND:**

Few studies have attempted to delineate the clinical profile of multiple Sclerosis (MS) among people of Asia. This study sought to identify the characteristics of early-onset Multiple Sclerosis (EOMS) comparison to adult-onset form (AOMS) in Isfahan, IRAN.

**METHODS:**

This prospective study was conducted on 104 youths with multiple sclerosis beginning before the age of 16 years and 123 patients with adult-onset multiple sclerosis. Patients were observed for a mean period of 5 years. The common presenting symptoms, MRI finding, course of disease and disability score were compared between the two groups.

**RESULTS:**

The mean onset age of disease in youths and adults were 14 ± 1.9 and 27.7 ± 8.06 years, respectively. Female/male ratio was 4.47:1 in EOMS and 3.92:1 in AOMS, this ratio was 7:1 in early childhood MS (≤ 10 year). The most common presenting symptom was optic neuritis in the EOMS group and paresthesia in AOMS. Optic neuritis was common in AOMS too, but brainstem/cerebellar signs were more common in EOMS than AOMS. Seizure occurred more frequently in EOMS than in the AOMS group (12.6% vs. 1.6%, respectively, p < 0.001). MRI showed that brainstem plaques were more prevalent in the EOMS compared with the AOMS group.

**CONCLUSIONS:**

It was concluded that early-onset MS does not significantly differ from adult form in terms of major clinical manifestation and course of disease, however Seizure is more common in EOMS, and brainstem and cerebellar symptoms as presenting symptom are more common.

Multiple Sclerosis (MS) is a chronic inflammatory demylinating and disabling disease, which primarily affect young adults between 20 and 40 years of age. In 0.4% to 10.5% of cases, onset occurs in childhood (before the age of 16 years).^1^,^2^

Although the onset of MS in childhood is recognized worldwide, several barriers exist to its prompt diagnosis in children.^3^ Many clinicians consider MS as an exclusively adult-onset disease. Therefore, they may not suggest such diagnosis in a child. Moreover, the clinical and radiographic diagnostic criteria for MS have not been validated clearly in the pediatric age group.^4^

As a result of variability of the clinical features and course and long term prognosis of early onset multiple sclerosis (EOMS), sometimes there is delay in diagnose and therapy of young patients.^5^,^6^

Natural history studies provide important information about MS course and prognosis that might help in treatment approaches.^7^,^8^ However, prognosis of MS according to its age of onset remains controversial. Some researchers reported better outcome in EOMS, whereas others found more favorable prognosis in AOMS than EOMS.^9^ It is suggested that although the course of MS might be slower in children compared to adults, significant disability may be accumulated in early adulthood and the patient will be disabled at a younger age.^10^ Nevertheless, some researchers found no influence of age of onset on the prognosis.^11^

Considering the importance of early treatment of MS in adult, the necessity of understanding the natural history of EOMS becomes more evident.^12^

In addition, understanding the differences between MS in children and adults is helpful, since research findings in adults could be extrapolated to children and teenagers.

The objective of this survey was to highlight the clinical and demographic features of EOMS in Isfahan, Iran and to compare the clinical and paraclinical features of EOMS with adult onset MS (AOMS).

## Methods

The sample of this study consisted of 106 MS patients in whom the first attack occurred before the age of 16 years. Simultaneously 130 clinically definite MS patients with adult onset disease and about same duration of disease randomly allocated from Isfahan MS clinics. Diagnosis of MS was confirmed by two neurologists according to McDonald criteria.^13^ Patients were monitored from January 2000 to August 2006 through routine clinical visits and regular telephone interviews.

All patients underwent brain MRI with field strengths of 1.0 Tesla superconductive system. Axial, sagittal, and coronal, T1-weighted, T2-weighted, fluid attenuated inversion recovery (FLAIR), and proton density sequence imaging were performed. MRI findings were analyzed by the Barkhof’s criteria.^14^

Two patients in EOMS and seven in AOMS were excluded from the study because follow-up was ceased.

### 

#### Data Collection

A clinical reporting form was designed for every patient and was used in the follow-up course.

The baseline data included familial MS history, age, sex and detailed clinical and MRI characteristics, seizure, and amount of disabilities in the beginning and after 5 years follow-up according to Kurtzke’s Expanded Disability Status Scale (EDSS),^15^ which was detected at least by two neurologists. Certain laboratory tests such as measurement of serum antinuclear antibodies (ANA), antineutrophil cytoplasmic antibodies (ANCA), antiphospholipids and anticardiolipin IgG and IgM antibodies, lupus anticoagulant were performed for all of patients to rule out common differential diagnosis of MS.

All MRI scans were reviewed by observers blinded to the clinical course.

The data collected for disease course included the characteristics of subsequent attacks and occurrence of any disability. Data were obtained directly from the medical records kept by the neurologists.

The mean duration of follow up was 4.9 years in EOMS patients and 5.1 years in AOMS.

Clinical course was classified according to standardized definitions.

The Ethics Committee of the Faculty of Medicine and Biomedical Sciences of the Isfahan University of Medical Sciences approved the study.

#### Statistical Analysis

The demographic and clinical data were stored in a database and were analyzed by SPSS version 11. Qualitative variables were compared using chi square test. Variables with p < 0.05 were considered significant.

## Results

Ultimately 227 MS patients were studied, of which, 104 patients had onset-age less than 16 years of age (EOMS) and 123 patients had onset in adult age (AOMS).

In total, the mean age at onset was 21.46 ± 9.1 years while it was 27.7 ± 8.06 (range: 19-40 years) in the AOMS group and 14 ± 1.9 (range: 5-16 years) in the EOMS group.

In the EOMS group 77.8% showed a clinical onset between 14 to 16 years; in 8 out of 104 patients (7.7%) the disease started at age 10 years or less. The latter group was considered as a true childhood-onset MS.

In EOMS group 81.7% (85 patients) and in AOMS group 79.7% (98 patients) were female (p = 0.696) and the female/male ratio was 4.47:1 vs. 3.92:1 in the two groups, respectively. The proportion of girl to boy was 7:1 in childhood-onset MS group, and female predominance was significant.

The most frequent clinical course in both MS groups was relapsing-remitting and 83.7% of EOMS and 84.6% of AOMS patients had this type of course (p = 0.85).

The most common presenting symptoms in EOMS patients were optic neuritis followed by brainstem/cerebellar symptoms, while they were paresthesia followed by optic neuritis and brainstem/cerebellar involvement in AOMS (Table 1). One of EOMS patient had encephalopathy picture at onset; the disease was repeated approximately 3 months later and was recovered, but after 5 months she had another relapse and got secondary progressive course.

**Table 1:** The Characteristics of patients in both MS groups

	AOMS n = 123	EOMS n = 104	P value
Mean age of onset	27.7 ± 8.06	14 ± 1.9	
Female/male (ratio)	98/25 (3.92/1)	85/19 (4.47/1)	
Disease course:			
Relapsing-remitting	104 (84.6%)	87 (83.7%)	0.85
Secondary progressive	16 (13.0%)	14 (13.5%)	0.92
Primary progressive	3 (2.4%)	3 (2.9%)	0.83
Optic neuritis at onset	36 (29.3%)	35 (33.7%)	0.48
Parasthesias at onset	45 (36.6%)	15 (15.4%)	0.9
Cerebellar/brainstem at onset	23 (18.7%)	32 (30.8%)	0.034
Motor at onset	18 (14.6%)	15 (14.4%)	0.9
Others at onset (seizure, sphincter symptom)	1 (0.8%)	4 (3.8%)	0.12
EDSS (after 5 years):			
< 3.5	104 (84.6%)	84 (80.8%)	0.46
≥ 3.5	19 (15.4%)	20 (19.2%)	
MRI finding:			
Periventricular	93 (75.6%)	77 (74%)	0.78
Cerebellar/brainstem	14 (11.4%)	18 (17.3%)	0.2
Seizure	2 (1.6%)	13 (12.6%)	0.00

Family history was investigated in all MS patients; 8.7% (n = 9 cases) of EOMS and 12.2% (n = 15 cases) of AOMS patients had a positive family history (p = 0.51). None of the cases of childhood MS patients reported a positive family history for MS.

In the brain MRI, periventricular plaque was the most frequent finding in both EOMS and AOMS groups. A high number of patients (74% in EOMS vs. 75.6% in AOMS) had periventricular white matter lesion compatible with MS plaques in T2 W and FLAIR imaging, without significant difference (p = 0.78). The most common finding of MRI in childhood-onset MS was also periventricular plaques (75%). Brainstem plaques were more frequent in the EOMS than in AOMS group, but the differences were not significant (17.3% vs. 11.4% respectively, p = 0.2).

Seizure occurred more frequently in EOMS than in the AOMS group (12.6% vs. 1.6% respectively, p < 0.001).

After 5 years follow up, 80.8 percent of the patients in the EOMS group and 84.6 percent of the patients in the AOMS group had EDSS of less than 3.5 with no significant difference (p = 0.46). One case of EOMS died after two years.

## Discussion

The present study showed that although, EOMS is less common, but it does not differ significantly from AOMS. However, some EOMS aspects are peculiar.

In the present series, the youngest age-onset was 5 years. The youngest known patient with MS was a 24 months old child reported by Bejar in 1984.^16^

It is well documented that MS is more common in women than men. According to the results of this study, the female/male ratio in the EOMS was 4.47:1 and in AOMS was 3.92:1, and 7:1 in childhood MS. These ratios are higher than many other studies, such as the one in Canada in which female to male ratio in childhood-onset MS was reported 3.2:1,^17^ and the one in Turkey which reported a ratio of 2.5:1.^10^ Several authors reported a higher frequency of MS among women especially during puberty age. In the study of Ghezzi et al female/male ratio was 4.7 in EOMS with age 12 years, suggesting role of hormonal changes in triggering MS onset.^18^ But in the present study, sex ratio in prepubertal period was not lower than pubertal period. So present findings may not support the role of hormonal changes in triggering MS.

Although the higher female to male ratio in childhood cases has been documented in some previous studies,^19^,^20^ the results of Simon et al showed significant male preponderance in the group age of less than 10 years.^2^

In the present study, the most common initial symptom was optic neuritis in EOMS and paresthesia in AOMS. Brainstem and cerebellar symptoms were significantly more common in EOMS and optic neuritis was common presentation in both groups. These findings agree with previous collaboration studies, which showed brainstem and cerebellar symptoms at onset were more common in EOMS patients.^5^

On the other hand, in a multicenter study of 125 patients with childhood MS, Duquette et al reported that sensory disturbances were the most common initial manifestation of disease occurring in 26.4% of cases, followed by optic neuritis.^19^ A cooperative retrospective study of 3375 MS patients (149 cases of EOMS) reported higher frequency of brainstem dysfunction in the EOMS group than in the AOMS group.^21^

Contrary to present findings, the most common presenting symptoms in Taiwan were limb weakness (62%) and visual disturbance (43%).^22^

Seizure was reported more frequent in MS patients than in general population and it has occurred in 10% of Indian multiple sclerotic patients.^23^ Overall seizure occurs in about 5% of children with MS, but it is much more common in children under age of 10 years.^3^,^24^,^25^ In the present study, 12.6% of EOMS had seizure that occurred in first two years of disease and 1.6% of AOMS had also seizure (p = 0.001). All of the patients had good prognosis and seizure was controlled completely by one antiepileptic drug, as in Striano et al study.^25^

Consistent with previous studies in EOMS, the clinical course was predominantly Relapsing-Remitting form (83.7%) and only 2.9% had primary progressive course.^19^

The family history was positive in 8.7% of EOMS and 12.2% of AOMS in the present study, while it was 13.2% in the study of Ozakbas et al.^11^ Periventricular plaques were common MRI findings in both groups, as it was in previous study and 90% of EOMS patients fulfilled Barkof’s criteria of MS. But brainstem plaques were more frequent in EOMS, which may explain more common brainstem finding in them.

There are several studies about the prognosis of EOMS patients.^26^ According to these studies after a long period of time from onset of the disease, clinical disabilities in EOMS are less than in AOMS, but ultimately clinical disabilities in EOMS reach a high degree in younger ages compared to the patients in the AOMS groups.^1^,^21^ The results of the present study showed no differences of disabilities in the two groups but a longer follow-up is necessary to decide about this point.

## Conclusions

The findings of this study suggest that EOMS should be similar to AOMS in many aspects but there are some differences such as more Brainstem/cerebellar signs at presentation and more seizure in course of disease, so paying attention to these symptoms is important.
